# Fetuin‐A (alpha 2HS glycoprotein) modulates growth, motility, invasion, and senescence in high‐grade astrocytomas

**DOI:** 10.1002/cam4.940

**Published:** 2016-11-23

**Authors:** Gladys N. Nangami, Amos M. Sakwe, Michael G. Izban, Tanu Rana, Philip E. Lammers, Portia Thomas, Zhenbang Chen, Josiah Ochieng

**Affiliations:** ^1^ Department of Biochemistry and Cancer BiologyMeharry Medical College1005 D.B. Todd Blvd.Nashville37208Tennessee; ^2^ Departments of PathologyMeharry Medical College1005 D.B. Todd Blvd.Nashville37208Tennessee; ^3^ Department of Internal MedicineMeharry Medical College1005 D.B. Todd Blvd.Nashville37208Tennessee

**Keywords:** Fetuin‐A, glioblastoma, growth, invasion, motility, senescence

## Abstract

Glioblastomas (high‐grade astrocytomas) are highly aggressive brain tumors with poor prognosis and limited treatment options. In the present studies, we have defined the role of fetuin‐A, a liver‐derived multifunctional serum protein, in the growth of an established glioblastoma cell line, LN229. We hereby demonstrate that these cells synthesize ectopic fetuin‐A which supports their growth in culture in the absence of serum. We have demonstrated that a panel of tissue microarray (TMA) of glioblastomas also express ectopic fetuin‐A. Knocking down fetuin‐A using shRNA approach in LN229, significantly reduced their in vitro growth as well as growth and invasion in vivo. The fetuin‐A knockdown subclones of LN229 (A and D) also had reduced motility and invasive capacity. Treatment of LN229 cells with asialofetuin (ASF), attenuated their uptake of labeled fetuin‐A, and induced senescence in them. Interestingly, the D subclone that had ~90% reduction in ectopic fetuin‐A, underwent senescence in serum‐free medium which was blunted in the presence of purified fetuin‐A. Uptake of labeled exosomes was attenuated in fetuin‐A knockdown subclones A and D. Taken together, the studies demonstrate the impact of fetuin‐A as significant node of growth, motility, and invasion signaling in glioblastomas that can be targeted for therapy.

## Introduction

Glioma is a devastating primary malignancy of the central nervous system. The median survival time is approximately 12 months for the most malignant form commonly known as glioblastoma (GBM) 1. The WHO classification of brain tumors is currently the accepted system 2. This includes a grading system that distinguishes four grades for astrocytomas: grades I, II, III, and IV. Typically, grades I and II are well differentiated and closely resemble astrocytes from which they originate. Higher grade tumors (III and IV) are considered anaplastic, showing increased cell density, cellular atypias, and increased mitotic activity 3. Grade IV astrocytoma is synonymous with GBM 2, 4. One of the most defining features of glioblastomas is robust angiogenesis, making them the most vascularized tumors in humans 5. Astrocytes together with oligodendrites make up the bulk of brain tumor microenvironments, with astrocytes accounting for almost 50% of the cells which normally surround brain tumors including those migrating to the brain from other parts of the body 6. Although the major signaling pathways that regulate growth, motility, and invasive capacities of high‐grade astrocytomas (GBM) have been extensively studied and reported 6, the potential role for fetuin‐A in the growth and invasive capacities of these brain tumors is yet to be appreciated and represents a significant gap in our knowledge.

Tumor cells generally have less requirement for serum supplementation in their growth media 7. We previously reported that the proliferation of head and neck squamous cell carcinoma cells in serum‐free medium relied on the ectopic fetuin‐A synthesized by these cells 8. Interestingly, we have demonstrated that fetuin‐A is the major growth‐promoting constituent of fetal bovine serum, particularly for tumor cell growth 9. In the present studies, we questioned whether growth of high‐grade astrocytoma cell lines under serum‐free conditions is also driven by ectopic fetuin‐A synthesized by the cells.

Fetuin‐A is a multifunctional protein whose major physiological role is the inhibition of ectopic calcification 10. It has also been implicated in metabolic syndromes such as diabetes where it modulates the insulin receptor thereby altering insulin sensitivity 11, 12 and myocardial infarction where it plays a role in the maturation of atherosclerotic plaques 13, 14. We reported that PyMT^+^ transgenic mice that also lacked fetuin‐A, had reduced mammary tumor incidence and prolonged tumor latency via the TGF‐beta signaling 15. Fetuin‐A is an established modulator of TGF‐beta signaling 16. More recently, studies have demonstrated that fetuin‐A is synthesized ectopically by a number of tumors including prostate 17, 18, pancreas 19, head and neck squamous cell carcinomas 8. A urinary peptide analysis to differentiate pancreatic cancer (PCA) from chronic pancreatitis (CP) revealed that fetuin‐A was the most prominent urinary peptide in PCA 20. Mechanistically, we have demonstrated that highly purified fetuin‐A not only activates PI3K/AKT as well as MAP kinase signaling in tumor cells 9 but it also mediates the biogenesis of tumor exosomes that are relevant in cell growth, motility, and invasion 21, 22.

In this report, we demonstrate that fetuin‐A is synthesized ectopically by high‐grade astrocytomas (GBM) as well as established GBM cell lines. Knocking down fetuin‐A in these cells reduced their growth as well as motility and invasive potentials both in vitro and in vivo. Fetuin‐A depleted glioblastoma cells growing in serum‐free medium, underwent senescence. In addition, inhibition of fetuin‐A uptake in GBM cells by asialofetuin‐A (ASF) induced senescence. Uptake of growth‐promoting exosomes was reduced in the fetuin‐A knocked down cells compared to the control cells.

## Materials and Methods

### Materials

Fetuin‐A (Pedersen fetuin‐A) and asialofetuin (ASF) were purchased from Sigma (St. Louis, MO). Fetuin‐A was purified as previously described 9. Senescence detection kit was purchased from Calbiochem /EMD Biosciences, Inc., San Diego, CA.

### Cells

The glioblastoma cell lines (LN229 and U‐138MG) were purchased from ATCC (Manassas, VA). To determine whether LN229 synthesize fetuin‐A, cells were grown in 10 cm dishes up to 70–90% confluency in complete DMEM/F12 medium containing 5% heat‐inactivated fetal bovine serum. Cells were then serum starved for 24 h, harvested by trypsinization, washed twice in PBS, and RNA was extracted using the Qiagen RNeasy kit (Valencia, CA). The Qiagen One‐step Platinum RT‐PCR (VWR) kit was used to generate cDNA. The primers (Invitrogen, Carlsbad, CA) were fetuin‐A (AHSG) sense (Fet1: 5′‐ CACAGAGGCAGCCAAGTGTA‐3′) and AHSG antisense (Fet2: 5′‐CTGGAGGAGCTGCCAGTAAC‐3′). PCR reactions were performed in duplicate. The PCR of cDNA from the mRNA protocol was 50°C for 50 min, 94°C for 2 min, 94°C for 30 sec, 55°C for 30 sec, 68°C for 1 min, and 72°C for 7 min for 35 cycles. Real‐Time PCR was accomplished with an IQ SYBR Green Supermix kit following the manufacturer's protocol. (Bio‐Rad, Hercules, CA). Copies of the targeted cDNA were normalized to *α*‐tubulin.

### Expression of fetuin‐A in brain TMA

TMA of normal brain parenchyma cells and astrocytomas tumors were purchased from US Biomax, Inc., Rockville, M.D. The microarray slides were deparaffinized using a standard xylene/ethanol/PBS protocol. Antigen retrieval was performed for 20 min at 98°C (60°C preheat/70°C cool down) using the Labvision PT module and PT Module citrate pH 6 Buffer (Thermo Scientific, Waltham, MA). Immunostaining was mechanically performed on the Labvision Autostainer using the Ultravision Quanto (HRP polymer) Detection System (Thermo Scientific). Standard incubation times were used except that the primary antibody was incubated for 60 min followed by a stringent 5‐min wash in TBS containing 0.1% Tween‐20. Monoclonal anti‐fetuin antibody (R&D Systems, Minneapolis, MN) was used at a 1:500 dilution in OP Quanto antibody diluent (Thermo Fisher, Waltham, MA). The Quanto DAB Plus system was used (5 min) for color development. The slides were counterstained with Mayer's hematoxylin (1 min), dehydrated, and coverslipped using cytoseal XYL (Thermo Scientific, Waltham, MA). Images were captured using a Nikon Eclipse E400 equipped with a Motic 5 MP digital webcam.

### In situ hybridizations

ISH was performed using the RNAscope 2.5 High Definition Assay ‐ BROWN (Advanced Cell Diagnostics, Hayward, CA) on brain primary tumor TMA GL208 (US Biomax, Inc.) or fixed cell pellet paraffin‐embedded slices of serum‐starved human glioblastoma cell line LN229. Manufacturer's instructions were followed, using RNA oligonucleotide probe cocktails that recognize mRNA of human AHSG (Hs‐AHSG), the positive control Hs‐PPIB housekeeping gene or as a negative control, the transcripts of the bacterial dapB gene product. Briefly, deparaffinized sections were hybridized to probes, followed by amplification by serial application of amplifiers, followed by peroxidase labels, and detection with DAB. 23. As recommended with low abundance mRNA pools, to enhance staining intensity when using Hs‐AHSG, we lengthened the incubation time with reagent Hybridize AMP 5 to 1 h. Images were obtained using an Olympus BX41 light microscope and captured with the Moticam 5.0MP digital camera.

### Knockdown of fetuin‐A in the glioblastoma cell line LN229

To knockdown fetuin‐A expression in LN229, cells were grown overnight in 6‐well plates until a density of 2 × 10^5^ cells per well in 2 mL of DMEM/F‐12, then transfected with scrambled control or two distinct human fetuin‐A (AHSG) shRNA in Lenti‐GFP using DNAfectin^™^ Plus, as per the manufacturer's protocol (Applied Biological Materials Inc., Richmond, BC, Canada). The cells were selected in complete medium containing 2.5 *μ*g/mL of puromycin for 1 month. Puromycin‐resistant and GFP‐positive cells were further isolated using fluorescence‐activated cell sorting (FACS) and thereafter maintained in selection medium as long as cells were in culture. Knockdown was validated by quantitative RT‐PCR and western blots.

### Motility and invasion assays

Scrambled shRNA‐transfected LN229 (SCR‐Control) as well as the two fetuin‐A knockdown subclones A and D were cultured until ~70% confluency in complete medium. The motility and invasion assays were performed as previously described 24. For the invasion assays, the upper Matrigel‐coated wells contained 2.5 × 10^5^ cells/well. The experiments were repeated three times.

### Growth and senescence assays

Cells (SCR‐Controls, A and D) were seeded in triplicates in 6‐well plates (1 × 10^4^ cells/well) containing either serum‐free or complete medium. Every other day for 8 days, the cells were trypsinized, and counted using hemocytometer and expressed as number of cells/mL. The growth curve experiments were repeated three times. Cells (SCR‐Controls and subclone D) were also seeded in SFM in 12‐well plates (2000 cells/well) and allowed to grow for 5 days. Since fetuin‐A was substantially knocked down in subclone D, the cells growing in some of the wells were supplemented with purified fetuin‐A (D + FetA) at 1 mg/mL. At the end of the incubation period, the cells were fixed and assayed for senescence‐associated beta‐galactosidase (SA‐*β*‐gal). Senescence Detection kit (Calbiochem) was used following the manufacturer's instructions. The number of senescent cells was expressed as percentage of total number of cells/20× microscopic field.

### The growth of scrambled controls of LN229 and its fetuin‐A knockdown subclones, A and D, in nude mice

To prepare the cells for in vivo bioluminescent imaging, we generated firefly luciferase expressing viruses by cotransfecting pLenti‐CMV‐V5‐Luc Blast (Adgene, plasmid # 21471) and lentivirus packaging mix (Applied Biological Materials) into 293T cells. Viruses were harvested 48 h posttransfection and concentrated using the ABM virus concentration reagent before use in transducing the glioblastoma cells. The transduced cells were selected using complete DMEM/F12 supplemented with 2.5 *μ*g/mL of blasticidin and 2.5 *μ*g/mL of puromycin. The cells were maintained in this selection medium. The cells were then injected (1 × 10^6^ cells/mouse) subcutaneously on both flanks on the dorsal surface of 6 weeks old J:Nu mice (Jackson Laboratories, Bar Harbor, ME), following our IACUC‐approved protocol. The mice were divided into three groups of eight mice/group. Group A (injected with SCR‐Control LN229 cells); Group B (subclone A cells); and Group C (subclone D cells). The growth of tumors in a representative mouse from each of the three groups was carefully followed and imaged on weeks 3, 4, and 5. The 21 remaining mice were also imaged at those time points and their tumor sizes were determined. Approximately, 10 min prior to imaging, the animals were injected with luciferin (i.p) and anesthetized with isoflurane as per the protocol. To quantify tumor sizes, we used a software associated with the imager (Carestream, Rochester, NY) that gives sum of intensities that is proportional to tumor size.

### Modulation of cell growth, uptake of labeled fetuin‐A, and motility in LN229 cells by ASF

LN229 cells were plated (2000 cells/well) in 24‐well plates in serum‐free medium in the absence (Control) or presence of ASF (0.5–1 mg/mL) and allowed to grow for 5 days. At the end of the incubation period, the cells were fixed and assayed for SA‐*β*‐galactosidase activities as described above. Purified fetuin‐A was also labeled with rhodamine isothiocyanate as previously described 25 and its uptake by subconfluent LN229 cells growing in SFM in glass chambered/welled slides examined in the absence (control) or in the presence of ASF (0.5–2 mg/mL) (Nikon A1R‐Confocal microscope). We questioned whether ASF also modulated the motility of LN229. Using Boyden chambers, motility in the presence of ASF (0–1 mg/mL) in the upper wells was done as described above. Lastly, we analyzed (western blots) the lysates of LN229 cells treated with ASF (0–1 mg/mL) to determine if it alters the levels of p53 in these cells 15.

### Uptake of GFP‐CD63‐labeled exosomes by LN229 subclones

It has been demonstrated that tumor cells endocytose/take up exosomes from the extracellular milieu and that this uptake mediates cellular adhesion, spreading, and motility 26, 27, 28. We questioned the abilities of glioblastoma cell lines expressing different quantities of fetuin‐A to endocytose or take up labeled exosomes. We previously demonstrated that one of the biological activities of fetuin‐A was its involvement in the uptake of exosomes by tumor cells 22. Exosomes were isolated and purified from BT‐CD63 breast carcinoma cells as previously described 21. The labeled exosomes were then added to naïve glioblastoma subclones in chambered glass slides in the presence of conditioned medium (containing fetuin‐A) from the three subclones of LN229 cells and incubated at 37°C for 1 h. The GFP‐CD63‐labeled exosomes were also added directly to A, D, and SCR‐Control subclones in SFM and incubated for 1 h. The chambers were removed and a drop of Slow Fade^™^ containing DAPI added to the slides, coverslipped, and prepared for confocal microscopy. Since the cells also contained GFP‐plasmids, the threshold settings for the cells prior to adding GFP‐exosomes, was set low to background levels. The cells that acquired exosomes from the extracellular milieu were quantified by counting the number of green cells in a 20× microscope field (Nikon A1R Confocal Microscope) and expressed as percentage of total number of cells (green+blue).

### Statistics

TMA data were analyzed at Vanderbilt University Digital Histology Shared Resource with Leica SCN400‐20× Bright‐field Scanning microscope. The images were analyzed with Digital Image Hub platform from Leica (Ireland). All data presented herein were analyzed by the GraphPad Prizm Statistical Package.

## Results

### Expression of fetuin‐A in grades of astrocytomas as revealed by immunohistochemistry of TMA sections

**Figure 1:**
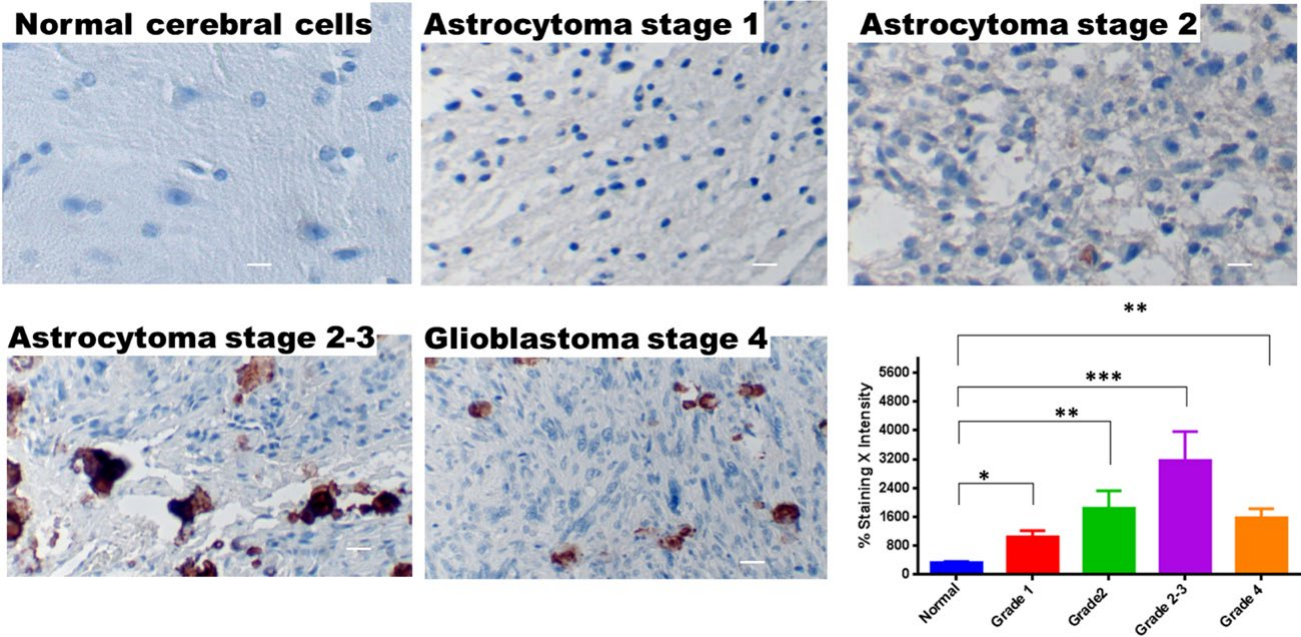
Expression of fetuin‐A in tissue microarrays of nomal cerebral cells and astrocytomas. The tissue microarrays were processed for immunohistochemistry as described in Materials and Methods, and stained with monoclonal antibodies to human fetuin‐A (ahsg). Normal brain cerebral cell arrays (*n* = 28); astrocytoma grade 1 (*n* = 24); astrocytoma grade 2 (*n* = 28); astrocytoma grade 2–3 (*n* = 33); and glioblastoma/astrocytoma grade 4 (*n* = 29) were analyzed by Digital Image Hub (Leica Systems). Percentage of staining per core was multiplied by staining intensity of that particular core. The bars represent mean ± SEM (*P < 0.05; **P < 0.01; ***P < 0.001). The scale bar represents 10 *μ*m

Fetuin‐A is expressed at negligible levels in normal brain cells (Fig. 1). However, it is expressed ectopically at high levels at specific hotspots of high‐grade astrocytomas and glioblastomas (Fig. 1). Astrocytoma (grades 2–3) showed the highest level of expression relative to normal brain cells (Fig. 1). The same tissues that showed high protein expression also showed ectopic fetuin‐A mRNA as revealed by in situ hybridization albeit not as obvious as protein levels. Fetuin‐A mRNA was depicted by small brown dots in the cytoplasm of cells that were absent in control slides (Fig. S1).

### Glioblastoma cells synthesize fetuin‐A that drives motility and invasion

**Figure 2:**
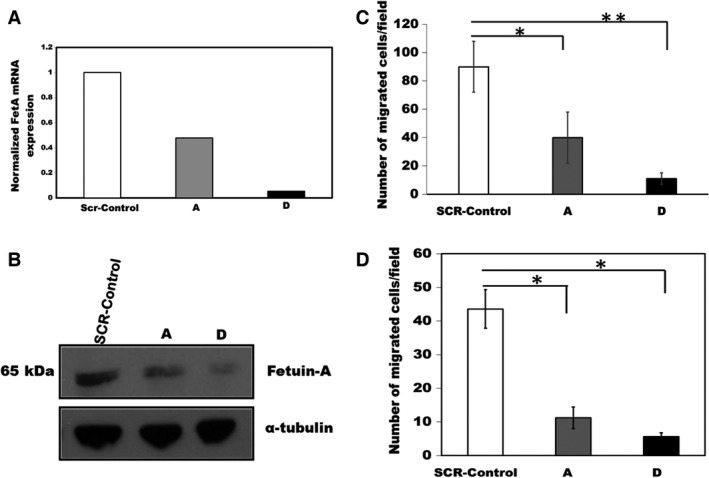
Synthesis and knockdown of fetuin‐A mRNA in LN229 and the associated motility and in invasion phenotypes. In panel (A), the expression of fetuin‐A mRNA in the SCR‐Control, and fetuin‐A knockdown cells (subclone A and D) was quantified by q‐RT‐PCR and normalized to *α*‐tubulin. In (B), cell lysates of the control and fetuin‐A knockdown subclones were probed for fetuin‐A expression (*β*‐actin for loading control) in western blot assays. The assays were repeated three times and gave similar data. In (C), the controls and fetuin‐A knockdown subclones were assayed for motility using Boyden chambers as described in Materials and Methods. The number of cells migrated to the underside of the polycarbonate were counted in the 20× objective field. The bars represent mean counts ± SEM of 30 fields from three independent experiments (*P = 0.01; **P = 0.001). In (D), the controls and fetuin‐A knockdown subclones were assayed for their invasion potential using Matrigel‐coated inserts as described in Materials and Methods and cells counted as in panel (C). The bars represent mean counts ± SEM of 30 microscopic fields from three independent experiments (*P < 0.0005).

We demonstrated by quantitative RT‐PCR that LN229 glioblastoma cells transcribe ectopic fetuin‐A mRNA (Fig. 2A) that is translated into protein (Fig. 2B). Interestingly, the fetuin‐A mRNA was recorded only in cells growing in serum‐free medium. In the presence of complete medium (10% serum), fetuin‐A synthesis was negligible (data not shown). Another glioblastoma cell line, U‐138MG, also synthesized fetuin‐A (data not shown). The synthesized human fetuin‐A (~65 kDa) has a higher molecular weight than liver‐derived protein that is approximately 48–55 kDa 29. We generated two LN229 subclones A and D in which fetuin‐A was knocked down by ~50% and ~90%, respectively (Fig. 2A). Of note was the reduced motility and invasive capacities (Fig. 2C and D, respectively) as some of the key biological consequences of knocking down fetuin‐A in LN229 cells.

### Tumor‐derived fetuin‐A promotes cell growth and attenuates senescence

**Figure 3:**
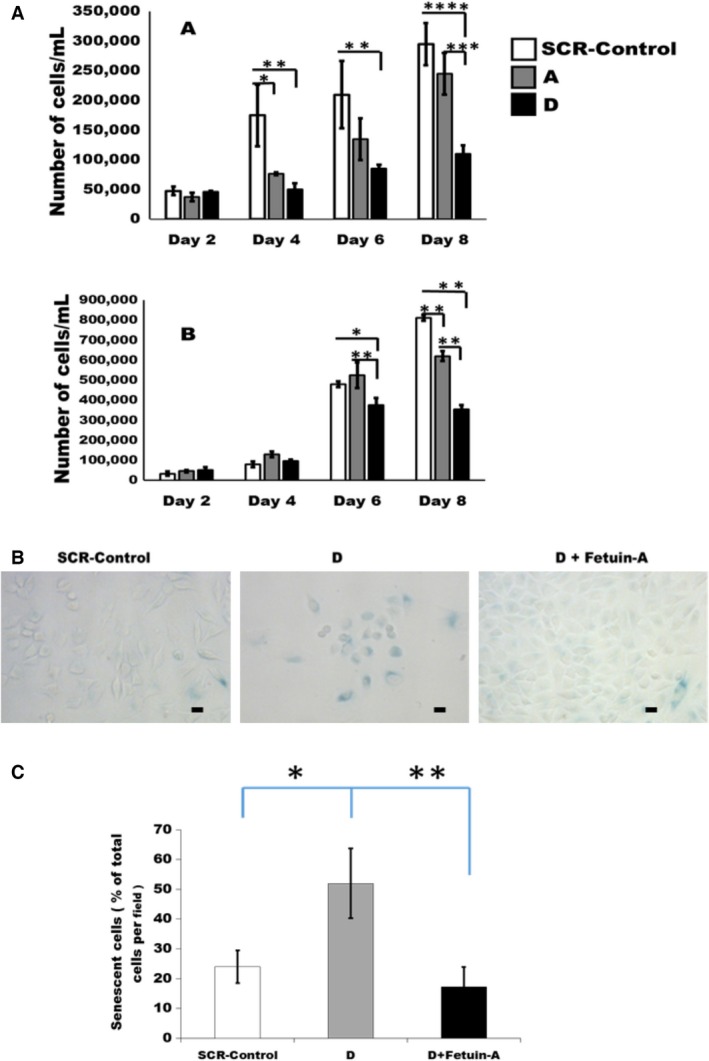
Growth curves and the emergence of senescent cells in the LN229 subclones. In (A), the cells were plated in triplicates in 6‐well plates at 1 × 10^4^ cells/well in either serum‐free medium (SFM) as shown in the upper panel (*P = 0.02; **P = 0.0014; ***P = 0.005; ****P = 0.0001), or complete DMEM/F12 medium containing 5% fetal bovine serum as shown in lower panel (*P = 0.002; **P = 0.0001); then beginning at day 2 postplating, the cells were dislodged using trypsin/EDTA and counted every other day using a hemocytometer for 8 days. The bars represent mean ± SEM of three separate experiments. In (B), SCR‐Control or D subclone cells were plated in 12‐well plates at 2000 cells/well in SFM and allowed to grow for 5 days. Some of the D subclones were seeded in SFM containing purified fetal bovine serum (1 mg/mL). At the end of the growth period, the cells were fixed in fixative solution and assayed for SA‐*β*‐galactosidase as described in Materials and Methods. The scale bar represents 10 *μ*m. In (C), dark blue senescent cells in panel (B) were counted in five 20× microscopic fields and expressed as average percentage of the total number of cells/20× field; 10 fields/data group. The bars represent % mean ± SEM of two separate experiments (*P = 0.01; **P = 0.005). SFM, serum‐free medium.

We consistently observed that parental LN229 and SCR‐Control cells, multiply exponentially and reach high cell densities in the absence of serum compared to the fetuin‐A knocked down sublines (A and D) as shown in Fig. 3A, upper panel. Interestingly, proliferation of the scrambled (SCR)‐Controls remained higher than the fetuin‐A knockdown subclones even in the presence of serum (Fig. 3A, lower panel). More importantly, a high percentage of D cells growing in serum‐free medium at confluence were much larger in size, suggesting the emergence of senescence phenotype 30. Indeed, SA‐*β*‐galactosidase assays revealed that ~50% of the subclone D had undergone senescence (Fig. 3B). Interestingly, addition of purified bovine fetuin‐A (1 mg/mL) to the SFM in which D cells were growing, reduced the percentage of senescent cells (Fig. 3C).

### Growth of glioblastoma control and fetuin‐A knocked down sublines as xenografts in nude mice

**Figure 4:**
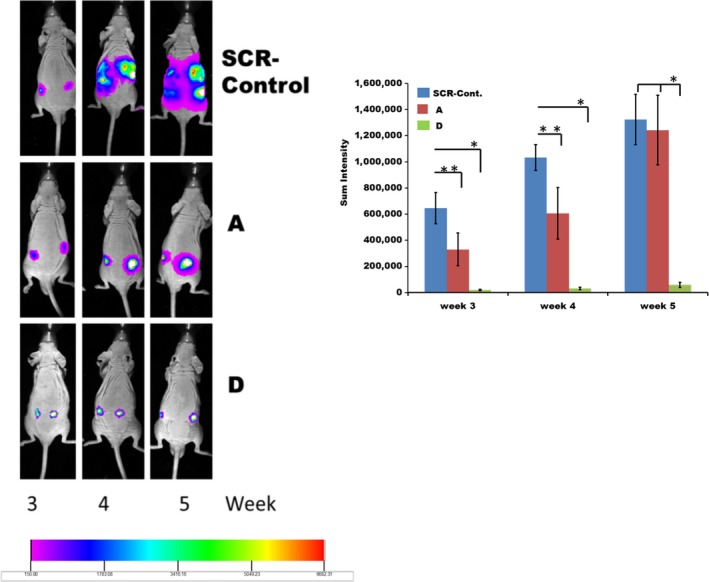
The in vivo growth of LN229 and its fetuin‐A knockdown subclones (A) and (D). The cells, SCR‐Controls, clones (A) and (D) expressing firefly luciferase were injected (1 × 10^6^ cells/mouse) subcutaneously on both flanks on the dorsal surface of 6 weeks old male J:Nu mice (Jackson Laboratories). The mice in each group were imaged on weeks 3, 4, and 5 after inoculation with the cells. The tumor growth in one representative mouse from each of the three groups was followed over the 5‐week period by imaging as shown. The areas (luminescence sum intensities) of tumor spread in all the 24 mice (*n* = 8 per group) were carefully measured and graphed as shown. The bars represent mean tumor size by luminescence intensity per group ± SEM. *P < 0.08; **P < 0.01

Glioblastomas are perhaps the most invasive tumors that do not colonize other organs away from the brain. We therefore wanted to determine the influence of fetuin‐A not only in the proliferation but more importantly the invasive capacities of glioblastoma cells in vivo. To allow the mice to live longer and reduce their suffering, we assessed the growth after subcutaneous rather than orthotopic implantation in the brain. As shown in Figure 4, by week 3, a number of the mice in group A (SCR‐Control) and group B (A subclone) had developed small palpable tumors. We therefore identified a mouse from each of the three groups that had the smallest “bump” and followed their tumor growth over the next 2 weeks. As can be seen, the representative mouse from group A had the largest tumors that had spread (invasive) extensively by weeks 4 and 5, respectively, followed by the group B representative. All the mice in groups A and B were euthanized by week 5 because the tumors growing on their backs reached the maximum threshold volume allowed by our protocol as shown by the Kaplan–Meier plot (Fig. S3). The tumor in group C (subclone D) representative mouse did not increase appreciably in size for the entire 5 weeks of follow‐up.

### Induction of senescence and abrogation of motility in LN229 cells by ASF

**Figure 5:**
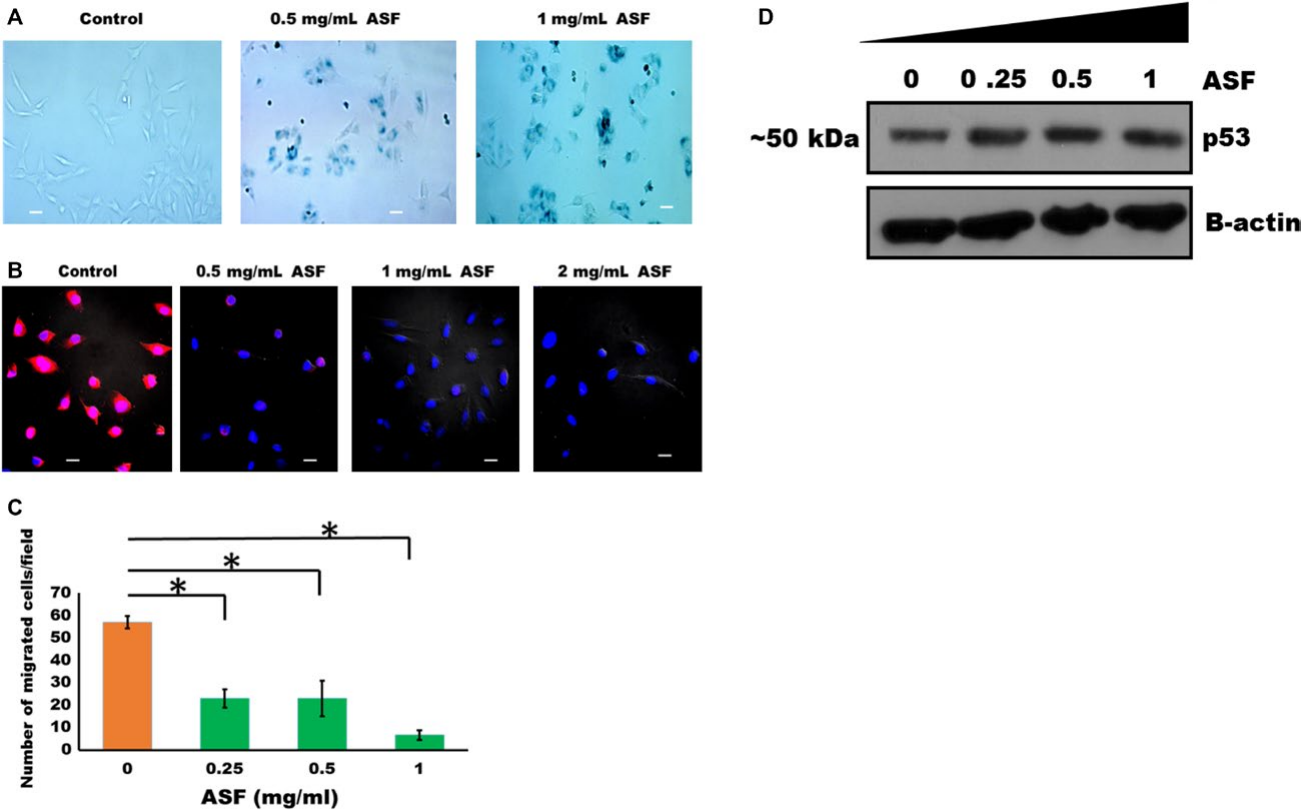
Asialofetuin‐A (ASF) induces senescence and attenuates uptake of labeled fetuin‐A and motility in LN229 cells. In (A), LN229 cells were plated (2000 cells/well) in 24‐well plates in serum‐free medium in the absence (Control) or presence of ASF (0.5 and 1 mg/mL) and incubated for 5 days. The cells were then fixed and assayed for SA‐*β*‐galactosidase (blue color). Senescence experiment was repeated a total of four times with similar data (bar represents 10 *μ*m). In (B), purified fetuin‐A was labeled with rhodamine isothiocyanate (pink color) and its uptake by LN229 cells growing in SFM in 4‐well glass slide examined in the absence (control) or in the presence of ASF (0.5–2 mg/mL) (Nikon A1R‐Confocal microscope). The uptake experiments were repeated a total of three times with similar data (bar represents 10 *μ*m). In (C), motility of LN229 cells in the absence or presence of ASF (0–1 mg/mL) was examined in Boyden chamber assays. The bars represent mean ± SEM of three separate experiments (*P = 0.0001). The LN229 cells were also treated with increasing concentrations of ASF (0–1 mg/mL) for 48 h, lysed in RIPA buffer containing protease inhibitors, resolved in SDS‐PAGE, transferred to nitrocellulose paper, and probed with antibodies to p53. The data shown in panel (D) is a representative of two different experiments. SFM, serum‐free medium.

We observed in four separate experiments that the growth of LN229 cells in SFM was significantly attenuated in the presence of ASF. Within 3 to 4 days in the presence of ASF, the cells became larger and stopped dividing. SA‐*β*‐galactosidase assays demonstrated that nearly all the cells in the presence of ASF (0.5 mg/mL and 1 mg/mL), underwent senescence within that period (Fig. 5A). More importantly, we observed that unlabeled ASF attenuated in a dose‐dependent manner the uptake of rhodamine isothiocyanate‐labeled fetuin‐A by LN229 cells (Fig. 5B). As additional control, the uptake of labeled fetuin‐A was only partially inhibited by the addition of unlabeled fetuin‐A (data not shown). Similar fetuin‐A uptake data were reported by others 31. Interestingly, the migration of LN229 was attenuated in the presence of ASF in Boyden chamber assays (Fig. 5C). We previously demonstrated that the uptake of fetuin‐A by cells is required for the generation of biologically active exosomes that in turn promote motility 21. Therefore, the abrogation of fetuin‐A uptake by ASF could explain its role in attenuating the motility process in these cells. Lastly, we questioned whether the senescence in LN229 cells induced by ASF was p53 dependent. Previous data showed that p53 was upregulated in the fetuin‐A null tumors 15. In the present studies, treatment of the glioblastoma cells with ASF which induces senescence, only marginally increased p53 (Fig. 5D), suggesting the contribution of other p53‐independent senescence pathways.

### Uptake of GFP‐CD63‐labeled exosomes is attenuated in fetuin‐A knocked down cells

**Figure 6:**
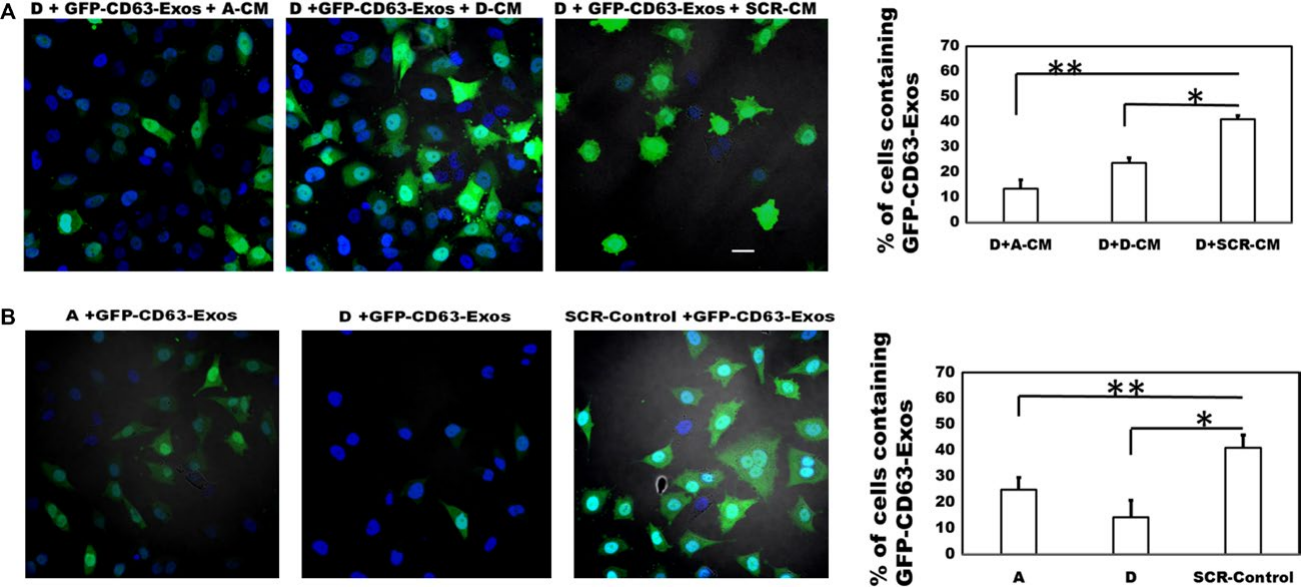
Fetuin‐A promotes the uptake of GFP‐CD63‐labeled exosomes in LN229 subclones. In (A), fetuin‐A knockdown subclone D cells were seeded at 1 × 10^4^ cells/well in three of the wells of a 4‐well glass slide. After an 8 h incubation in SFM, the medium in the wells was replaced with previously collected conditioned medium (1 mg/mL; 200 *μ*L/well) from fetuin‐A knockdown subclone (A) (A‐CM); subclone (D) (D‐CM), and SCR‐Control (SCR‐CM) cells. After setting the GFP‐channel to the lowest threshold, labeled exosomes (isolated and purified from BT‐CD63; 20 *μ*g/mL) were then added to each well (30 *μ*L/well) and incubated for 1 h. The cells were fixed in 4% formalin (w/v), a drop of slow fade with DAPI added to each slide, coverslipped, and examined under a Nikon A1R confocal microscope. The number of green cells (containing exosomes) in a 20× microscopic field (five different fields/data point), were counted and expressed as percentage of total cells (green+blue) per field. The bars represent mean ± SEM of two separate experiments (*P = 0.0003; **P = 0.0001). In (B), the LN229 subclones (A, D, and SCR‐Control) were seeded in three of the wells of a 4‐well glass slide as above in SFM and incubated for 8 h. After adjusting the GFP‐fluorescence channel to the lowest threshold as in (A) above, the GFP‐labeled exosomes (20 *μ*g/mL) were added to each of the three wells and incubated for 1 h, cells fixed in 4% formalin, and prepared as above for confocal microscopy. The percentage of green cells was determined as above and the bars represent mean ± SEM of two separate experiments (*P = 0.0002; **P = 0.0004; scale bar represents 10 *μ*m). SFM, serum‐free medium.

We previously demonstrated that fetuin‐A in the extracellular milieu promotes rapid uptake of exosomes by cultured tumor cells 22. In the present studies, we questioned whether exosomal uptake is attenuated in fetuin‐A knocked down glioblastoma cells. Uptake of exosomes is essential for adhesion, cell spreading, and motility 26. The fetuin‐A synthesized by the glioblastoma cells is secreted into the conditioned medium (data not shown). The data show that although conditioned media from the A and D subclones marginally promoted the uptake of GFP‐CD63‐labeled exosomes by D cells, conditioned medium from SCR‐Control cells, with most of the fetuin‐A, displayed optimal uptake of labeled exosomes (Fig. 6A). Similarly, the uptake of GFP‐CD63‐labeled exosomes was high in SCR‐Control cells as expected, but attenuated in A and D cells in SFM (Fig. 6B). The experiment was repeated one more time and data collected as shown in Figure 6.

## Discussion

In the present studies, we have demonstrated the significance and impact of ectopic fetuin‐A synthesized by astrocytomas for their growth and progression. The potential role of fetuin‐A in the progression of astrocytomas has essentially been overlooked. A report by Petrik et al., indicated that preoperative serum levels of fetuin‐A (AHSG) was low in most patients with astrocytomas 32. Their data demonstrated that ~10–20% of GBM patients had normal serum fetuin‐A levels (~0.4 mg/mL) and that this was associated with prolonged survival. It is unclear whether ectopic fetuin‐A synthesized by GBM can modify the levels of serum AHSG (liver derived). Based on the data reported herein, it is likely that low serum fetuin‐A levels (~<0.3 mg/mL) switches on the synthesis of ectopic fetuin‐A which is more efficient at promoting growth in the glioblastomas. Normal serum fetuin‐A levels (~0.4 mg/mL) on the other hand suppress the synthesis of ectopic fetuin‐A, thereby slowing down tumor progression. Another likely scenario is that ectopic fetuin‐A is synthesized mainly at the onset of astrocytoma transformation and just before extensive neovascularization. As the astrocytoma tumors grow larger and become more vascularized, they obtain and consume more fetuin‐A from the blood supply resulting in low serum levels that translates to poor prognosis.

The appearance of the senescence phenotype in LN229‐clone D (reduced fetuin‐A synthesis), growing in serum‐free medium, was in agreement with our earlier studies in which we reported that a high percentage of mammary tumor cells in fetuin‐A null transgenic PyMT C57BL/6 mice was senescent 15. Thus, our studies suggest that one of the key roles of fetuin‐A, is the inhibition of senescence signaling pathways in tumors. The uptake of fetuin‐A either by autocrine or paracrine mechanisms is necessary as a prelude to the degradation of p53 15 and other pathways that promote tumor growth instead of senescence 33. Just like apoptosis, senescence is a major tumor suppression pathway both in vitro and in vivo 34, 35. The data suggest that the extremely slow growth of the fetuin‐A knockdown GBM xenograft (clone D) 5 weeks after inoculation was due to senescence. However, once angiogenesis was set particularly in the control group and clone A that had ~50% reduction in the ectopic fetuin‐A synthesis, tumor growth was accelerated.

Previous report from our laboratory indicated that fetuin‐A denuded of its sialic acid residues lacked the capacity to support cellular adhesion 36. Interestingly, for fetuin‐A to promote adhesion, it has to be internalized by the cells. Cells that lack the capacity to take up fetuin‐A adhere poorly 9. The ability of asialofetuin‐A to competitively inhibit the uptake of fetuin‐A suggest two basic mechanisms: (1) that both fetuin‐A and asialofetuin‐A are internalized via the same endocytic pathway and (2) that asialofetuin‐A assumes the role of dominant negative inhibitor of fetuin‐A‐mediated physiological functions such antisenescence and promotion of cellular motility. Unfortunately, asialofetuin is relatively unstable in vivo 37 and cannot be used effectively to abrogate the tumor‐promoting functions of fetuin‐A.

Mechanistically, studies suggest that fetuin‐A transmits its tumor growth, motility, and invasive signals via activated exosomes or oncosomes 21, 22. Fetuin‐A obtained from serum or synthesized and secreted ectopically by the tumor cells, is endocytosed and while inside the tumor cells, it binds to histones, which it loads on exosomes to activate them. Sialic acid residues on fetuin‐A are required for exosomal loading because asialofetuin‐A which is taken up via a similar route as fetuin‐A and can also bind to histones intracellularly, lacks the ability to interact with exosomes (Fig. S2), and consequently has no biological activity. The present data support the notion that binding of fetuin‐A‐activated exosomes to their putative receptors on the cell surface transmits adhesion, motility, invasion, and growth signals in tumor cells 26, 27, 38. The rapid in vivo growth of control LN229 cells also suggest that the secreted bioactive exosomes modified the tumor microenvironment by promoting angiogenesis 39, 40. This ensured the recruitment of more fetuin‐A (liver derived) and other growth factors that accelerated the growth further.

It should be emphasized that there is considerable molecular and clinical heterogeneity in GBM suggesting the presence of multiple molecular phenotypes. For this reason, it is important to uncover a pathway that encompasses most if not all the molecular subtypes and that can be targeted for therapy. We have demonstrated that ectopic fetuin‐A (tumor derived) promotes the growth, motility, and invasive potentials of LN229 glioblastoma cells. Ectopic fetuin‐A was also synthesized by a panel of high‐grade astrocytoma tumor cells. More importantly, the studies suggest that one of the major roles of fetuin‐A is to attenuate senescence that slows down growth, motility, and invasion potentials in glioblastoma cells. Lastly, we have demonstrated that sialic acid residues on fetuin‐A are essential for its ability to promote the progression glioblastomas and that externally added asialofetuin‐A acts as a dominant negative form of fetuin‐A. Taken together, fetuin‐A uptake mechanisms of glioblastoma cells can be targeted to inhibit the growth of this aggressive brain tumor.

## Conflict of Interest

None declared.

## Supporting information


**Figure S1.** In situ hybridization to determine the level of fetuin‐A mRNA in astrocytoma stage 2–3. The in situ hybridization was performed as described in Materials and Methods using RNAscope kit from Advanced Cell Diagnostics, Hayward, CA. Fetuin‐A mRNA appear as brown dots (arrows) in the cytoplasm.

**Figure S2.** Uptake of fetuin‐A and asialofetuin (ASF)‐coated exosomes by LN229 glioblastoma cells. Exosomes were isolated and purified from LN229 cells as described (20, 21, 28). The exosomes (20 *μ*g/mL) was divided into two equal portions of 100 *μ*L each. One was incubated with 100 *μ*L rhodamine isothiocyanate‐labeled fetuin‐A (1 mg/mL), while the other with 100 *μ*L of rhodamine isothiocyanate‐labeled ASF for exactly 1 h. The exosomes were then washed 3X, and after each wash, centrifuging them at 100,000*g* for 2 h. The final precipitate was dissolved in 100 *μ*L of Hanks‐buffered saline containing 1 mmol/L of Ca^2+^ ions. The exosomes were then added to LN229 plated on glass coverslips and allowed to incubate for 2 h. The cells were washed twice with Hanks‐buffered saline, coverslips with cells placed on a drop of slow fade with DAPI, and examined under a confocal microscope. The precipitated exosomes coated with fetuin‐A were red, while ASF‐coated exosomes yielded a white precipitate indicating that labeled ASF did not associate with the exosomes.

**Figure S3.** Survival curves of nude mice inoculated with LN229 and its fetuin‐A knockdown subclones. Mice that had tumor volumes of 15 mm^3^ were removed from the study and euthanized as per our protocol. By week 4, two of the eight mice were removed for euthanization from the scrambled control group (group A) and one of the eight mice inoculated with subclone A cells (group B) was euthanized. By week 5, the remaining mice in groups A and B were euthanized due to tumor size. Only mice inoculated with subclone D cells survived the duration of the study.
